# Sequence-Specific Binding of Recombinant Zbed4 to DNA: Insights into Zbed4 Participation in Gene Transcription and Its Association with Other Proteins

**DOI:** 10.1371/journal.pone.0035317

**Published:** 2012-05-31

**Authors:** Vladislav V. Mokhonov, Veena P. Theendakara, Yekaterina E. Gribanova, Novruz B. Ahmedli, Debora B. Farber

**Affiliations:** 1 Jules Stein Eye Institute and Department of Ophthalmology, David Geffen School of Medicine, University of California Los Angeles, Los Angeles, California, United States of America; 2 Molecular Biology Institute, University of California Los Angeles, Los Angeles, California, United States of America; 3 Brain Research Institute, University of California Los Angeles, Los Angeles, California, United States of America; Center for Genomic Regulation, Spain

## Abstract

Zbed4, a member of the BED subclass of Zinc-finger proteins, is expressed in cone photoreceptors and glial Müller cells of human retina whereas it is only present in Müller cells of mouse retina. To characterize structural and functional properties of Zbed4, enough amounts of purified protein were needed. Thus, recombinant Zbed4 was expressed in *E. coli* and its refolding conditions optimized for the production of homogenous and functionally active protein. Zbed4’s secondary structure, determined by circular dichroism spectroscopy, showed that this protein contains 32% α-helices, 18% β-sheets, 20% turns and 30% unordered structures. CASTing was used to identify the target sites of Zbed4 in DNA. The majority of the DNA fragments obtained contained poly-Gs and some of them had, in addition, the core signature of GC boxes; a few clones had only GC-boxes. With electrophoretic mobility shift assays we demonstrated that Zbed4 binds both not only to DNA and but also to RNA oligonucleotides with very high affinity, interacting with poly-G tracts that have a minimum of 5 Gs; its binding to and GC-box consensus sequences. However, the latter binding depends on the GC-box flanking nucleotides. We also found that Zbed4 interacts in Y79 retinoblastoma cells with nuclear and cytoplasmic proteins Scaffold Attachment Factor B1 (SAFB1), estrogen receptor alpha (ERα), and cellular myosin 9 (MYH9), as shown with immunoprecipitation and mass spectrometry studies as well as gel overlay assays. In addition, immunostaining corroborated the co-localization of Zbed4 with these proteins. Most importantly, *in vitro* experiments using constructs containing promoters of genes directing expression of the luciferase gene, showed that Zbed4 transactivates the transcription of those promoters with poly-G tracts.

## Introduction

Zinc-finger proteins constitute 2–3% of the entire human genome [1], and are related to a wide range of biological functions such as development, differentiation, mRNA trafficking, cell adhesion, cytoskeleton organization, and suppression of tumors [2]. Zinc-fingers are the most common DNA-binding motifs found in human transcription factors [1]. They have been divided into several classes according to the number and type of amino acids involved in Zn^2+^ coordination, such as C_2_H_2_, C_2_HC, C_4_ ribbon, C_4_ GATA family, C_6_, C_8_, C_3_HC_4_ ring finger, and H_2_C_2_ [3]–[6]. In addition to binding DNA, zinc-finger domains are now recognized to bind RNA, protein and/or lipid substrates [7]. Their binding properties depend not only on the amino acid sequence of the fingers but also on that of the linker between fingers, the number of fingers and the higher-order structures.

One subclass of zinc-finger proteins has the BED finger DNA-binding domain, characterized by the signature Cx_2_Cx_n_Hx_3-5_[H/C] (x_n_ is a variable spacer) and the presence of two highly conserved aromatic amino acids (tryptophan and phenylalanine) at its N terminus [8]. The BED finger is found in proteins of plants (the tobacco 3AF1 [9] and tomato E4/E8-BP1 [10]) and animals [the *Caenorhabditis elegans* Dpy-20 protein [11] in one or more copies. BED fingers are also found in proteins like the AC1 and Hobo-like transposases [8]. At least 6 ZBED human proteins (ZBED1-ZBED6) belong to the subclass of BED zinc-finger proteins [12]–[15]. These proteins are thought to function as either transcription activators or repressors by modifying local chromatin structure on binding to GC-rich sequences [8].

ZBED4 is a recently described protein [14], [16]. Its amino acid sequence contains 4 BED zinc-finger domains and two LXXLL nuclear receptor-interacting modules in the N-terminal half, and an hACT dimerization domain in the C-terminal half. In addition to its BED finger-related hypothesized function as a DNA-binding protein, ZBED4 could also act as a co-activator/co-repressor of nuclear hormone receptors through its LXXLL motifs [17]. In human retina, ZBED4 is localized to both the nuclei of cone photoreceptors and the cytoplasm of their inner segments and pedicles, as well as to glial Müller cells' endfeet [14]. Interestingly, in mouse retina Zbed4 is detected only in Müller cells and their processes, but not in cones [16].

The role of ZBED4 in subcellular compartments of cells is still not known. In this study, we provide initial structural and functional characterization of mouse Zbed4. We successfully developed a high-level expression system for the production of recombinant Zbed4 in *E. coli* and an efficient method for the refolding of the protein after purification under denaturing conditions. We then used circular dichroism spectroscopy (CD) to study the secondary structure of Zbed4, and characterized its DNA- and RNA-binding ability *in vitro* and the nature of the sites to which it binds. We also found that Zbed4 interacts with nuclear and cytoplasmic proteins and corroborated these results by co-immunoprecipitating Zbed4 from Y79 retinoblastoma cells with the identified proteins, Scaffold Attachment Factor B1 (SAFB1), Estrogen Receptor alpha (ERα) and cellular Myosin 9 (MYH9). Most important, our studies show that Zbed4 functions as a transcription factor, positively affecting the transcriptional activity from G-rich promoters of genes expressed in retina.

## Results

### Expression and Refolding of Zbed4

**Figure 1:**
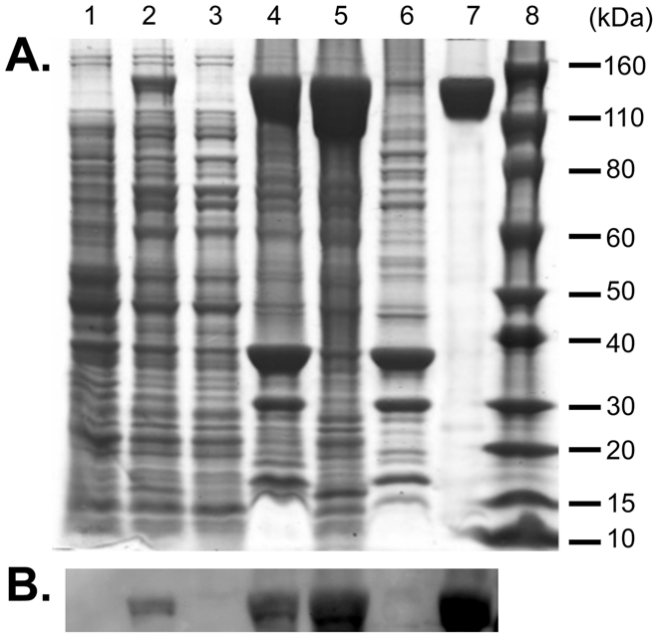
Fractionation and purification of recombinant Zbed4 protein expressed in *E. coli* cells. **A.** SDS-PAGE. 50 µg/well of total protein from each fraction obtained in the expression, purification and refolding of Zbed4 were separated by SDS-PAGE on 4–12% Bis-Tris gels and stained with Coomassie R-250. *Lane 1,* whole *E. coli* lysate before IPTG induction. *Lane 2,* whole cell lysate after 6 h induction by IPTG. *Lane 3,* soluble proteins of *E. coli* cell lysate in HEPES buffer containing 1% Triton X100 and other components (see Materials and Methods), after passing through a French pressure chamber and centrifugation at 150,000 g. *Lane 4*, insoluble material (inclusion bodies) of lysate. *Lane 5*, solubilized inclusion bodies in 6 M Gu-HCl buffer 1, after centrifugation at 150,000 g. *Lane 6*, insoluble fraction of inclusion bodies. *Lane 7*, Zbed4 purified using BD Talon Co^2+^-activated affinity chromatography, after the refolding procedure and concentration. *Lane 8*, Novex Sharp (Invitrogen) standard protein markers. **B.** Detection of Zbed4 on Western blots using Penta His antibodies. Following SDS-PAGE, the separated proteins of each fraction were transferred to PVDF membranes and after blocking and incubation with Penta His antibodies conjugated with horseradish peroxidase, Zbed4 was visualized with the ECL Substrate of the Fast Western blot kit.

The expression level of a protein varies as much as 2- to 5-fold among different strains of *E. coli* [18], so for expression of Zbed4 we initially tested several strains of *E. coli*: BL21(DE3), BL21(DE3)Star, B834(DE3), C41(DE3), and C43(DE3) (Table S1). All these strains are deficient in the OmpT and Lon proteases that could interfere with the isolation of intact recombinant proteins, carry the lambda DE3 lysogen that encodes T7 RNA polymerase, and are designed for high-level expression of proteins [19]. None of them, except for BL21(DE3)Star, produced any trace of Zbed4, probably due to the toxic effect of the high level of protein expressed, which could cause a cellular response to the foreign recombinant molecules and degradation of heterologous mRNA by host RNases. Since *E. coli* BL21(DE3)Star cells lack RNaseE activity, mRNA degradation does not occur in them. Thus, we co-expressed Zbed4 in these cells with the pLysSRARE2 plasmid, which encodes T7 lysozyme and rare codons necessary for the synthesis of eukaryotic proteins in *E. coli* and prevents leaky basal expression [20]. This approach allowed us to obtain Zbed4 in inclusion bodies. The formation of inclusion bodies depends on the rate of protein synthesis and folding [18]. In an attempt to avoid their formation, we tested different growth temperatures (37^o^, 25^o^ and 4°C) and IPTG concentrations (1, 0.5, and 0.1 mM). However, no effects on the solubility of Zbed4 were observed even at 4°C and lowest IPTG concentration. We found that the maximum amount of Zbed4 obtained was about 30% of the total protein in the original lysate when *E. coli* cells were grown at 37°C and induced by 0.5 mM IPTG. These conditions were chosen for expression of our target protein (Fig. 1, lanes 1, 2, 3 and 4). After solubilization of Zbed4 from the inclusion bodies (Fig. 1, lanes 5 and 6) and purification using metal affinity chromatography, we obtained ∼ 20 mg of denatured His-tagged Zbed4 protein from 3.8 g of wet *E. coli* pelleted cells (1 L culture).

The choice of buffer during the refolding process is important, so we tried different buffer systems including Tris, MOPS, HEPES, PIPES, TRICINE and BICINE. The optimum pH range for refolding is between 7 and 8 since lower pH leads to re-aggregation of recombinant proteins. Addition of Zn^2+^ ions is necessary for the correct conformation of the zinc-finger motifs in the DNA binding domain of Zbed4. We also tested addition of various agents (MgSO_4_, CHAPS, L-glutamine/L-arginine, ascorbic acid, propyl gallate) and methods of refolding (dialysis, chromatography or slow dilution) to improve the refolding efficiency. We found that serial steps of dialysis against refolding buffer (see Materials and Methods) at 4°C were optimal. Coomassie blue R-250 staining of the SDS-PAGE separated proteins and Western blot analysis showed that the refolded protein was ∼ 90% pure (Fig. 1, lane 7). The purified and refolded protein was subsequently used for CD spectroscopy and DNA-binding analyses.

### Prediction and Experimental Determination of the Secondary Structure of Zbed4

The secondary structures in proteins do not conform to a single geometry. Deviations from ideal conformational angles lead to distortions in the secondary structure such as bends, twists, etc. The protein CD spectrum is an average of CD signals from all conformations.

**Figure 2:**
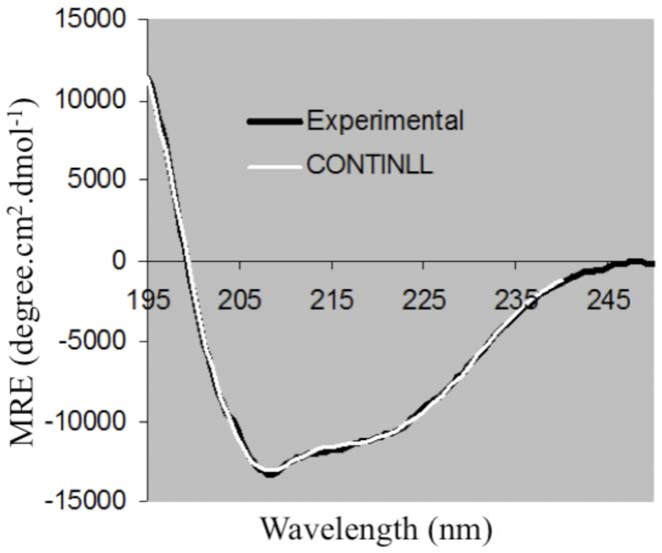
Far-UV CD spectrum of Zbed4 (black curve) and curve obtained using the CONTINLL program (white curve). The CONTINLL-calculated curve conforms well to the experimental spectra of Zbed4. SELCON and CDSSTR-calculated curves (not shown) were essentially identical to that of CONTINLL.

The far-UV CD spectrum of Zbed4 (0.4 mg/ml) exhibits two distinct minima, one around 208 nm and a shoulder with a minimum around 220–222 nm (Fig. 2), which are indicative of α–helix conformation [21]. For quantitative estimation of the fractional content of secondary structures in Zbed4 [α-helices, β-strands, turns and random coil (unordered)] this spectrum was analyzed using the three programs in CDPro software (http://lamar.colostate.edu/sreeram/CDPro/main.html). CDPro is a series of programs for protein analysis that contains three popular CD calculation programs, CONTINLL, SELCON3, and CDSSTR [22]. These programs are based on a locally linearized ridge regression method (CONTINLL), and the self-consistent method using the singular-value decomposition algorithm (CDSSTR and SELCON3). The three programs use the reference set of proteins SMP56, which includes the CD spectra of 13 membrane and 43 soluble proteins. The curve estimated for Zbed4 by CONTINLL using SMP56 as the reference set is also shown in Figure 2. Both experimental and calculated curves correlate very well and are essentially superimposable. Practically the same curves were obtained using CDSSTR (not shown), and SELCON3 (not shown).

Table S2 represents the estimated fractional content of secondary structure for Zbed4 at pH 7.3. It shows that the three programs, CONTINLL, SELCON3 and CDSSTR gave similar results.

The normalized root-mean-square deviation (nrmsd) value was used to judge the goodness of the fit between the experimental spectrum and the curve calculated from the crystallographic data in SMP56.

The nrmsd was calculated as: 
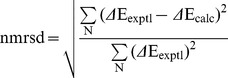
 where ΔE_exptl_ and ΔE_calc_ are the experimental and calculated dichroic molar absorption per amino acid residue, respectively, and N is the number of data points.

The nmrst values for CONTINLL and CDSSTR are 0.018 and 0.080, respectively, which indicate excellent agreement between estimated and calculated curves. The nmrst value for SELCON3 is relatively high, 0.349. However, the results obtained using SELCON3 are very consistent with those of CONTINLL and CDSSTR. Therefore, we averaged the results of the three programs: Zbed4 is estimated to have 32.8% α-helices, 17.7% β-strands, 20% turns and 29% unordered structures.

For predicting the theoretical secondary structure based on the amino acid sequence of Zbed4 we selected the Garnier-Robson algorithm [23], based on the fact that this method provided very consistent results for all 6 human ZBED protein sequences in terms of % α helices, β-strands, turns and unordered structures. This algorithm is available from the FASTA Sequence Comparison web resource at the University of Virginia (http://fasta.bioch.virginia.edu/fasta_www2/fasta_wwwcgi?rm=misc1) and the DNASTAR Lasergene software (DNASTAR, Madison, WI). Similar results to the averaged values of the three programs in CDPro were obtained by using the Garnier-Robson algorithm for the prediction of theoretical secondary structure of Zbed4∶38% α-helices, 18% β-strands, 24% turns and 20% unordered structures.

The number of α-helices and β-strands in Zbed4 can be calculated using the CDPro results. CONTINLL, CDSSTR, and SELCON3 divide the α-helix and β-strand conformations into “regular” and “distorted” fractions. The distorted fractions of the secondary structure elements reflect the fact that a certain number of the end-residues of α-helices and β-strand fragments do not have a regular hydrogen bonding. It has been shown that, on average, four residues per α-helix and two residues per β-strand are distorted [24]. Consequently, the number of α-helices and β-strands can be estimated using the following formulas: 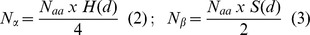 where N_aa_ is the total number of amino acid residues in the protein, and H(d) and S(d) are the distorted fractions of the α-helix and β-strand conformations, respectively.

**Figure 3:**
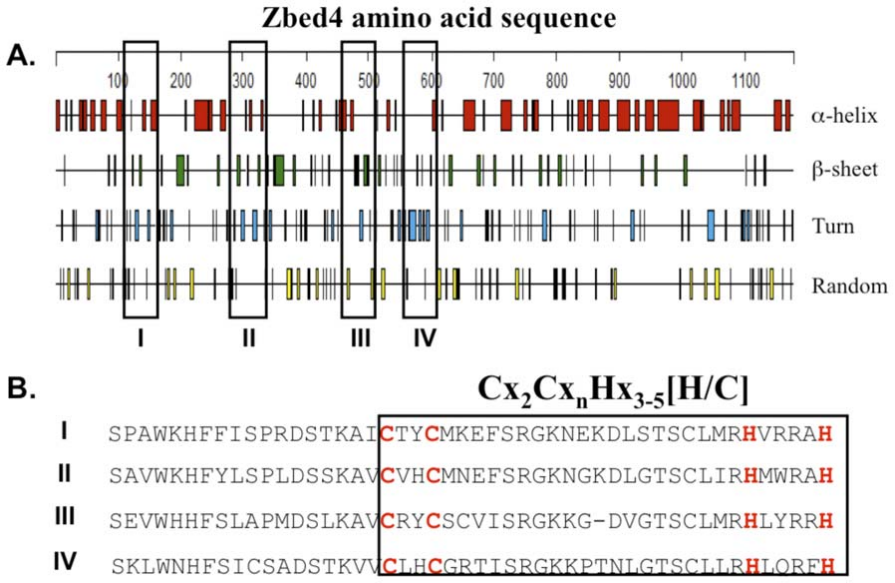
Prediction of secondary structures for Zbed4 using the Garnier-Robson algorithm. **A**. Position of α-helices, β-sheets, turns and random amino acid sequences in the Zbed4 1,168 amino acid sequence. Predictions performed by two different programs, DNASTAR (Lasergene) and the FASTA Sequence Comparison web resource at the University of Virginia, gave the same results. Boxes I, II, III and IV indicate the position of BED zinc-finger motifs in Zbed4. **B**. The amino acid sequence of the four boxes in **A** show the specific signature of the BED-type zinc-fingers present in Zbed4 containing the characteristic cystines and histidines (red).

We estimated the number of α-helical and β-strand segments in the Zbed4 sequence by introducing into formulas 2 and 3 the fractional content of distorted α-helical [H(d)] and β-strand [S(d)] structures from Table S2. This gave us an estimate of 42 α-helices and 45 β-strands distributed along the 1,168 amino acids of the Zbed4 sequence. These results correlate well with the predicted values obtained using the Garnier-Robson algorithm from the DNASTAR and FASTA comparison softwares (Fig. 3A), which indicate that Zbed4 has 49 α-helices and 51 β-strands along its 1,168 amino acid sequence. In Figure 3B, we are showing the sequence of the four-BED Zinc- fingers of ZBED4. The BED finger signatures are boxed.

### Identification of Zbed4 Binding Sites in Nucleic Acids using Cyclic Amplification and Selection of Targets (CASTing)

**Figure 4:**
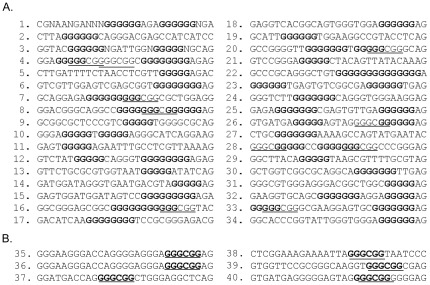
Nucleotide sequence of DNA fragments selected by the CASTing method. dsDNA (CASTrandom oligonucleotide, Table S3, after synthesis of the complementary strand using PCR-specific primers CAST-F and CAST-R) was incubated with Zbed4 to form protein-DNA complexes that were immunoprecipitated using Zbed4 antibody and protein A-Sepharose beads. Bound DNA was extracted, PCR-amplified and used for the next round of CASTing. Three rounds of CASTing were performed. The amplified DNA fragments that interacted with Zbed4 were cloned and sequenced. **A**. Clones carrying poly-G tracts (bolded) and GC-box core sequences (underlined). **B**. Clones only carrying GC-box core sequences.

Nucleic acid ligands with high affinity to Zbed4 were isolated by CASTing. This method involves alternate cycles of ligand selection from pools of random sequences and amplification of the ligand that binds to the protein of interest [25]. We synthesized an 84-base oligonucleotide containing a random set of 26 bases (making this a 4^26^-fold degenerate oligonucleotide) flanked by restriction sites and PCR priming sequences (Table S3, #9, CASTrandom oligonucleotide). After synthesis of the complementary strand using PCR-specific primers (Table S3, primers CAST-F and CAST-R), the double-stranded oligomer was mixed with the bacterially expressed Zbed4 to permit the formation of protein-DNA complexes that were then immunoprecipitated with protein A and Zbed4 antibody. Unbound DNA was removed by washing, and bound DNA was amplified by PCR. Several cycles of immunoprecipitation and PCR amplification were performed. The obtained DNA segments were cloned into pCR2.1-TOPO. Figure 4A lists the sequences of 34 of these clones, all of them containing poly-Gs. Some of these clones also contained the core signature of GC-boxes (underlined). In addition, six other clones were identified carrying only the GC-box sequence (Fig. 4B). These results indicated that Zbed4 has high affinity for poly-Gs and GC-boxes.

**Figure 5:**
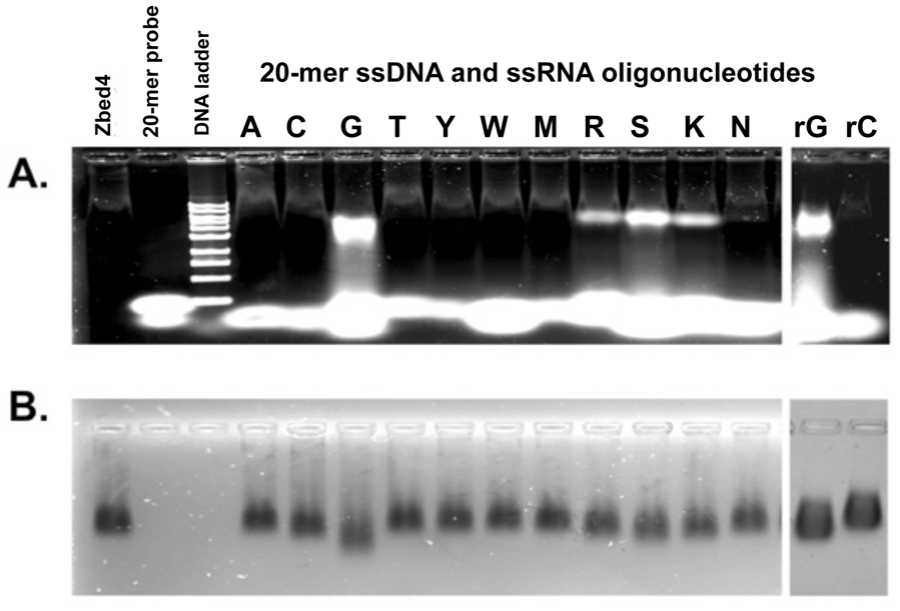
Relative affinity of recombinant Zbed4 for the different oligonucleotides. Zbed4 (20 µg) was incubated with 0.5 nM different 20-mer ssDNA (left panel) and ssRNA oligonucleotides (right panel) for 45 min and the whole reaction mixtures (20 µl) with the protein-DNA complexes were subjected to EMSA on 1% agarose gels run at room temperature in HEPES buffer, pH 8.2, at 20 mA. **A**. Agarose gel stained using SYBR Gold for detection of nucleic acid retardation. **B.** The same gel stained with Coomassie R-250 for detection of Zbed4 protein. Zbed4 (20 µg) and a single primer (0.5 nM) were applied separately to the gel as controls. 1 kb DNA ladder was used as a standard for nucleic acid size. As seen, Zbed4 binds only to DNA and RNA 20-mers that contain poly-Gs.

In order to determine the relative affinity of recombinant Zbed4 for the different nucleotides, we first designed degenerative 20-mer oligonucleotides (Table S3, primers #12 to 22). These degenerative primers were used together with Zbed4 in an electrophoretic mobility shift assay on agarose gels. When the gels were stained with SYBR Gold for the detection of nucleic acids (Fig. 5A), they showed that Zbed4 bound oligonucleotides that contained guanylyl residues, such as poly-G, poly-R, poly-S and poly-K (Table S3, primers #14, 19, 20 and 21). No signal was observed with poly-A, poly-C and poly-T, or with poly-Y, poly-W, poly-M and poly-N (Table S3, primers #12, 13, 15, 16, 17, 18 and 22). The same gel stained with Coomassie R-250 for the detection of protein (Fig. 5B) showed that Zbed4 was present in all lanes except in those containing a 20-mer primer, used as control, and the DNA ladder.

Being that some zinc-finger proteins can bind RNA molecules in a specific manner [7], we tested the binding ability of Zbed4 for RNA oligonucleotides. We synthesized 20-base-long ssRNA oligonucleotides containing 10 ribo-Gs or 10 ribo-Cs flanked by 5 Us at the 5′ and 3′ ends (Table S3, primers #42 and 43). Zbed4 efficiently interacted with the poly-ribo-G oligonucleotide, but no signal was detected with the poly-ribo-C probe (Fig. 5A). These data suggest that Zbed4 binds effectively both DNA and RNA and this binding is sequence-specific.

**Figure 6:**
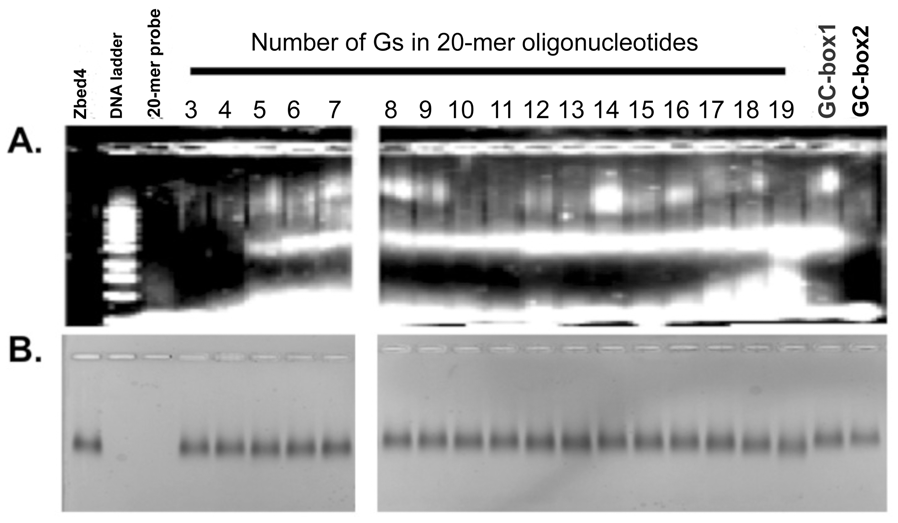
Determination of the minimal poly-G tract required for interaction with Zbed4 and of the affinity of Zbed4 for two different GC-box consensus sequences. Zbed4 (20 µg) was incubated with each of different 20-mer ssDNA primers containing G-tracts flanked by a different number of poly-As (0.5 nM), and with each of two GC-box consensus sequences for 45 min. The 20 µl reaction mixtures were loaded on a 1% agarose gel and EMSA was carried out as in Figure 5. A single primer (0.5 nM) and Zbed4 (20 µg) were used as controls. 1 kb DNA ladder was used as a standard for nucleic acid size. A. Agarose gel stained with SYBR Gold. B. The same gel stained with Coomassie R-250. As seen, the affinity of Zbed4 binds to for the oligonucleotides increases with the increasing number of Gs in the tracks. that contain at least 5 Gs and Quantification of the Zbed4-oligonucleotide complex bands showed that those with 5–11 Gs, 12–16 Gs and 17–18 Gs have similar density values: 105,415±15,217; 167,810±13,671; and 214,727±15,861, respectively. The complex with 19Gs has the highest value: 360,835. In addition, Zbed4 binds only to the GC-box, GGGGCGGGGC, indicating that the neighboring nucleotides of the core sequence are critical.

Next, we determined the minimal poly-G tract that would bind to Zbed4. Oligonucleotides with a different number of poly-G tracts were used (Table S3, primers #25 to 41). Zbed4-DNA interaction was observed with probes that contained at least 5 consecutive Gs (Fig. 6A); we also used EMSA to determine the affinity of Zbed4 for two GC-box consensus sequences, G**GGGCGG**GGC or T**GGGCGG**AAT ([26] and Table S3, primers #23 and 24) and found that Zbed4 formed a complex only with GC-box1 (Fig. 6A). Figure 6B, stained for protein, shows Zbed4 in every lane except for those containing the DNA ladder and a 20-mer primer used as control.

### Identification of Proteins from Y79 Retinoblastoma Cells that Interact with Zbed4

We used immunoprecipitation assays and mass spectrometry to search for Zbed4-interacting proteins. Cell extracts were incubated with Zbed4 antibody or pre-immune serum and the precipitated proteins were then subjected to SDS-PAGE and stained with SYPRO Ruby. Five protein bands that were not detected in the separated proteins resulting from the reaction with pre-immune serum were excised and destained. After *in situ* digestion with trypsin of the proteins in each band, the resulting peptides were subjected to nLC-MS/MS mass spectrometry**.** Analyses of the data obtained using the Sequest database search algorithm from the proteome Discoverer software, identified several proteins from each band, some of them corresponding to peptides with very high hits. In addition to ZBED4, nuclear SAFB1 (131 hits) and cytoplasmic MYH9 (112 hits) were among those proteins with highest scores.

### In vitro and In vivo Confirmation of the Zbed4 and SAFB1 Interaction

**Figure 7:**
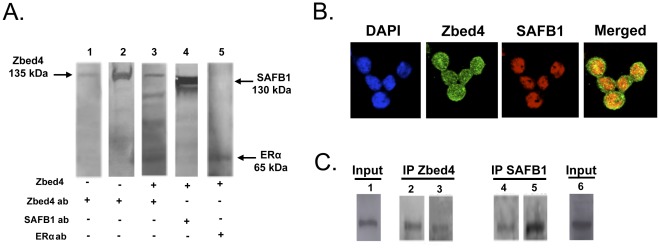
Zbed4 interacts with SAFB1 *in vitro* and *in vivo*. **A.** Membrane overlay assay: Proteins were resolved on SDS-PAGE and transferred to a PVDF membrane. Lane 1: cell lysate; lane 2: purified Zbed4 protein; lanes 3, 4 and 5: cell lysate incubated with Zbed4 protein (overlay). Lanes 1, 2 and 3 were probed with the Zbed4 antibody and lanes 4 and 5 with the SAFB1 and ERα antibodies, respectively. **B.** *Subcellular co-localization of Zbed4 with SAFB1*, carried out as described in Materials and Methods. The merged image clearly shows that Zbed4 co-localizes with SAFB1 in the nucleus of Y79 retinoblastoma cells. DAPI was used to stain the nuclei. **C.** Co-immunoprecipitation experiments were performed using Y79 retinoblastoma cell extracts. Both Zbed4 and SAFB1 were detected in the immunoprecipitated proteins obtained with Zbed4 antibody (left panel) or SAFB1 antibody (right panelIn each case, each duplicate lane of the blots obtained after SDS-PAGE of the immunoprecipitated proteins was incubated with Zbed4 or SAFB1 antibodies. Immunoprecipitation experiments were performed using Y79 retinoblastoma cell extracts (Input) and antibodies against Zbed4 or SAFB1. Duplicate aliquots of the proteins immunoprecipitated by each antibody were immunoblotted after SDS-PAGE with antibodies against Zbed4 (lanes 2 and 4) or SAFB1 (lanes 3 and 5). Both Zbed4 and SAFB1 are detected in each immunoprecipitate. Aliquots of Input material also show on the Western blots the presence of Zbed4 (lane 1) and SAFB1 (lane 6).

To analyze the *in vitro* interaction between Zbed4 and SAFB1 we used the membrane overlay assay. Figure 7A shows the presence in the cell extract of endogenous Zbed4 (lane1) at ∼135 kDa, corroborated by the position of purified Zbed4 run on the same gel (lane 2). Lane 3 shows that after overlaying with Zbed4 and washing extensively the membrane, the Zbed4 antibody recognized several bands indicating that Zbed4 had bound other proteins of the nuclear extract. One of these proteins with an apparent MW of 130 kDa was confirmed to be SAFB1 by probing a Zbed4-overlayed lane with a SAFB1 antibody (lane 4). Thus, this *in vitro* assay provided evidence for Zbed4 and SAFB1 interaction and confirmed the mass spectrometry results.

To evaluate the *in vivo* interaction of native Zbed4 and SAFB1 proteins, we first used immuno-cytochemistry and confocal microscopy imaging to investigate whether they co-localized in Y79 retinoblastoma cells. These cells, which contain a thin cytoplasm that surrounds a very large nucleus, were immunostained with anti-Zbed4 − Alexa 488 and anti-SAFB1 − Alexa 568 fluorescent antibodies. Figure 7B shows that the two proteins are present in the nucleus of Y79 cells, as it would be predicted if they are part of the same molecular complex. In addition, the merged image shows that Zbed4 is also present in the cells’ cytoplasm, around the nucleus (green staining).

We also carried out co-immunoprecipitations using Y79 retinoblastoma cells’ nuclear extracts and antibodies against Zbed4 or SAFB1. Lanes 2 and 3 of Figure 7C show the proteins precipitated with the Zbed4 antibody and immunoblotted after SDS-PAGE with the same antibody (Zbed4, lane 2) or the SAFB1 antibody (SAFB1, lane 3). Both Zbed4 and SAFB1 are found in the Zbed4 immunoprecipitate. A reverse experiment using the same nuclear extract and the SAFB1 antibody also showed the presence of both Zbed4 and SAFB1 in the SAFB1 immunoprecipitate, corroborating their *in vivo* interaction in Y79 retinoblastoma cells.

### Interaction and Co-localization of Zbed4 with ERα and MYH9

**Figure 8:**
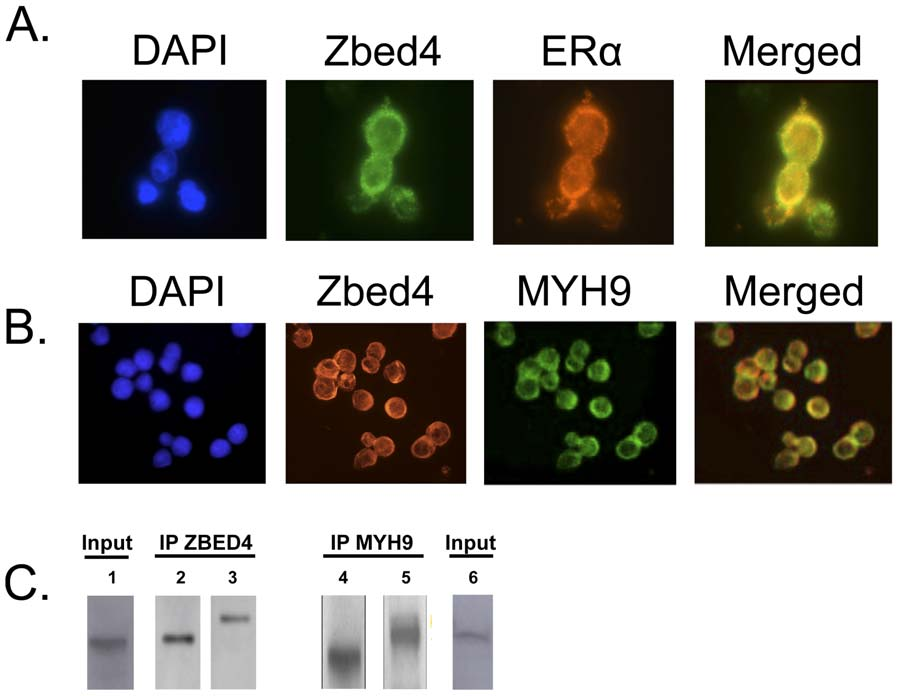
Co-localization of Zbed4 with ERα and MYH9 in Y79 retinoblastoma cells, carried out as described in Materials and Methods. The immunostaining results indicate that both ERα and MYH9 co-localize with Zbed4 in these cells. The major difference is that in A co-localization is seen in the nuclei and cytoplasm for Zbed4 and ERα whereas in B it is only observed in the cytoplasm for Zbed4 and MYH9. C. Immunoprecipitation experiments were carried out as in Figure 7C with Y79 retinoblastoma cells (Input, lanes 1 and 6) but using antibodies against Zbed4 (lanes 1, 2 and 4) or MYH9 (lanes 3, 5 and 6) instead; both Zbed4 and MYH9 are detected in each immunoprecipitate.

SAFB1 is a potent ERα co-repressor. Since Zbed4 interacts with SAFB1, it could be part of the SAFB1−ERα complex that results in ERα transcriptional activation of several genes. However, Zbed4’s nuclear receptor-interacting modules (LXXLL) that are characteristic of co-activators/co-repressors of nuclear hormone receptors, could also bind directly to ERα. We investigated this possibility carrying out similar experiments to those described above for Zbed4 and SAFB1. The membrane overlay assay showed that after overlaying Zbed4 on the lane containing the separated proteins from Y79 retinoblastoma cells and probing the membrane with an ERα antibody, ERα was clearly detected (Fig. 7A, lane 5). These results demonstrated that, indeed, Zbed4 and the ∼65 kDa ERα were in the same overlay membrane. Furthermore, immunocytochemistry showed that Zbed4 and ERα co-localize in both the nuclei and cytoplasm of Y79 retinoblastoma cells (Fig. 8A, Merged image). Similar experiments also demonstrated that Zbed4 and MYH9 co-localize to the cytoplasm of these cells and both proteins co-immunoprecipitate after incubation with the Zbed4 or MYH9 antibodies (Figs. 8B and 8C).

### Zbed4 Binding Sites in the 5′-flanking Region of Genes

**Figure 9:**
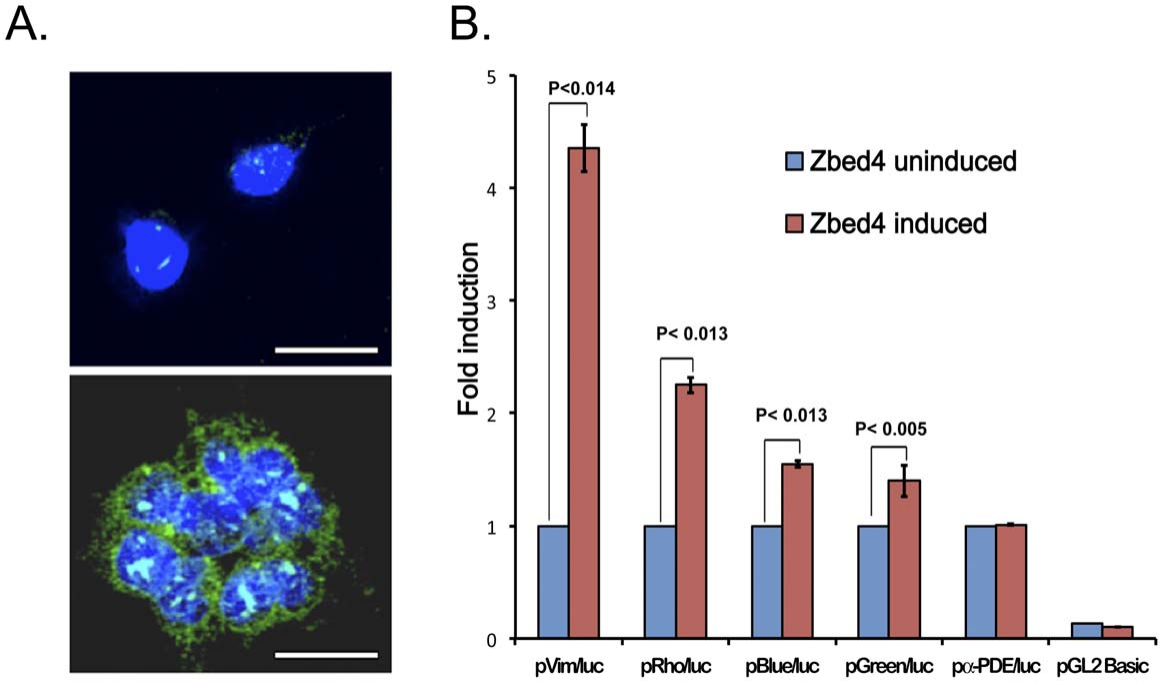
Zbed4 mediates transcriptional activation of several genes expressed in retina. **A.** Immunocytochemical detection of endogenous Zbed4 in HEK293 cells (upper image) and in HEK293 cells stably transfected with a Zbed4 expression construct (lower image). The endogenous Zbed4 in HEK293 cells is localized mainly in the nuclei (cyan) and barely detected in the cytoplasm whereas in the HEK293 stable-transfected cells Zbed4 is seen in both, the nuclei (cyan) and cytoplasm (green). DAPI was used to stain nuclei. **B**. Trans-activation of promoters from genes expressed in retina by Zbed4. Stably transfected HEK 293 cells expressing Zbed4 and non-transfected HEK 293 cells were used in transient transfections with luciferase reporter constructs carrying different retinal promoters. Luciferase activity was measured in the cell lysates and normalized for each transfection system to the corresponding β-galactosidase activity for each sample. The results are expressed as fold induction by Zbed4 of the mean luciferase activity of the uninduced promoter compared to that of the Vim/*luc*, Rho/*luc,* Blue/*luc*, Green/*luc*, and α-PDE/*luc* reporter constructs, respectively, ± S.D. p values for each pair of Zbed4 stimulated and non-stimulated promoters are noted above the bars.

To verify whether Zbed4 expressed in mammalian cells was able to interact with G-rich DNA sequences in a manner comparable to its recombinant prokaryotic counterpart, and to test Zbed4’s ability to activate/repress transcription *in vivo*, we used stable transfected cells expressing Zbed4 and reporter plasmids containing the promoter region of genes that have poly-G tracts in a luciferase assay. HEK293 cells stably transfected with pZbed4eu (Table S1) and expressing full length Zbed4, and non-transfected HEK293 cells were used for this experiment (Fig. 9A)**.** Considering that Zbed4 binds to poly−G tracts, we decided to test Zbed4’s effect on sequences of four genes that contained at least 5 consecutive Gs in their 5'-flanking region: mouse vimentin (two 5G-tracts, at −979 to −975 and −925 to −921), bovine rhodopsin (one 8G-tract, at −20 to −13), and human blue (one 6G-tract, at −8 to −3) and green (one 5G-tract, at −123 to −129) opsins. The appropriate promoter region for each gene was subcloned into the pGL2 Basic vector (Table S1). The rhodopsin reporter plasmid [27] was kindly provided by Dr. Donald Zack (Departments of Molecular Biology and Genetics, and Neuroscience, Johns Hopkins University School of Medicine, Baltimore, Maryland). As controls, we used the empty pGL2 Basic vector and a region of the non-specific α-PDE promoter (which contains no G-tracts), subcloned into the same vector.

Zbed4 expressed in the stable transfected HEK293 cells increased the luciferase activity produced by pVim/*luc*, pRho/*luc*, pBlue/*luc* and pGreen/*luc* above the expression level observed with non-transfected HEK293 cells, used as control, but Zbed4 did not have an effect on the α-PDE/*luc* construct (Fig. 9B). The highest increase was seen with the vimentin 5'-flanking region, followed by the 5′-flanking regions of rhodopsin, blue opsin and green opsin. The differences in promoter-driven luciferase activity between Zbed4 stable-transfected and non-transfected HEK293 cells were reproducible in three different experiments carried out in triplicate, and are statistically significant (p<0.014 for vimentin, p<0.013 for rhodopsin, p<0.013 for blue opsin and p<0.005 for green opsin, Student’s t-test). These results suggest that Zbed4 binds *in vivo* to and positively regulates the transcriptional activity of promoters containing poly-G tracts.

## Discussion

In this paper we describe the purification, refolding and expression of the mouse protein, Zbed4, and analyze some of its structural and functional properties. As we show, Zbed4 is able to bind DNA and RNA sequences and to trans-activate transcription of several genes.

To obtain the recombinant protein in amounts sufficient for further investigations, we first examined the effects of temperature and IPTG concentration on the induction of Zbed4 synthesis in *E.* *coli* cells. No target protein was detectable in cells that had not been exposed to IPTG, and we found that the optimal conditions, those that gave us the highest yield of protein, were a temperature of 37°C and an IPTG concentration of 0.5 mM. The yield decreased dramatically when lower temperatures were used, independent of the IPTG concentration.

The solubility of the *E. coli*-expressed protein was also investigated. After centrifugation of the whole cell lysate, Zbed4 was found only in the pellet (Fig. 1), indicating that the recombinant protein was being expressed in the bacterial cells as inclusion bodies. We could not extract Zbed4 from the inclusion bodies using 8 M urea, but another chaotropic agent, 6 M guanidine HCl, allowed us to solubilize the denatured protein, which was then purified using cobalt affinity chromatography.

Different approaches have been used for the refolding of denatured proteins (direct dilution [28], [29], chromatographic methods [30], [31], etc.). For us, several rounds of dialysis against refolding buffer were optimal to refold the denatured Zbed4 to its native state and activity. Other denatured zinc finger proteins had been successfully refolded in this way [32] **.** We also observed that the efficiency of refolding largely depended on the initial protein concentration: for Zbed4, 0.6 mg/ml worked very well. At higher concentrations, there was aggregation during dialysis. In addition, refolding strongly depended on the presence of Zn^2+^ ions, which are necessary for the correct conformation of the zinc fingers and for protein stabilization [33]–[35], and a reducing agent to reduce non-native inter- and intramolecular disulfide bonds and to keep the cysteines in their reduced state [36]. In our experiments we used 10 µM ZnSO_4_, and 5 mMβ-mercaptoethanol.

**Figure 10:**
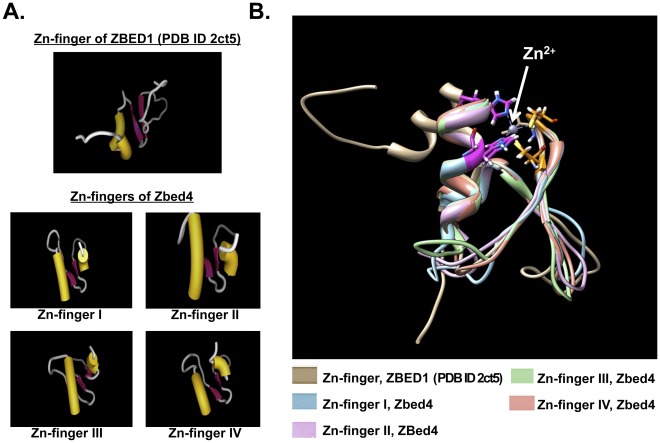
A. Structure modeling for BED zinc-finger (I, II, III and IV) domains of Zbed4, based on the structure of ZBED1 (PDB ID: 2ct5). The program iMol, version 0.40 and the UCSF Chimera package [39] were used to generate these models. **B**. Superimposed model of all predicted BED zinc-finger structures of Zbed4 (I, II, III and IV) and ZBED1 (PDB ID: 2ct5). Each finger is shown in a different color.

The far-UV CD spectrum of a protein generally reflects its secondary structure content. In the case of Zbed4, it indicated that the protein is primarily organized in an α-helix structure. Three different programs, CONTINLL, SELCON3 and CDSSTR, used to quantify the content of secondary structures in Zbed4 at pH 7.3, gave results in the same range, and thus, the results were averaged. The α-helix value obtained (32.8%) is very similar to the α-helix content calculated for most zinc-finger proteins including ZBED proteins [37] **.** Furthermore, the Garnier-Robson algorithm for the prediction of theoretical secondary structure based on the amino acid sequence of Zbed4 also provided similar results to those obtained using the data of the CD spectrum: 38% α-helices, 18% β-strands, 24% turns and 20% unordered. The Garnier-Robson program also allowed us to find out where the α-helices and β-strands are in the Zbed4 sequence. Figure 3A shows the distribution of these structures as well as the turns and unordered regions of the protein. It also shows the location of the 4 zinc-BED fingers. In addition, in Figure 3B the BED zinc-finger specific signature is shown. Moreover, we have generated 3D structural models for these BED zinc-fingers (Fig. 10A). The 3D structure of macromolecules offers insights into their molecular function at the atomic level, and often it provides direct evidence of molecular interactions between individual macromolecules, or between macromolecules and small molecules. By standard sequence similarity searches close to 60% of protein sequences can be mapped to a 3D structure [38]. We took the same sequence similarity approach when creating the 3D structure of all four BED zinc-domains of Zbed4 based on the solution structure of the zinc-finger BED domain-containing protein 1 (Zbed1, PDB ID: 2ct5), using the display application for sequence and structure information, Chimera [39]. Our models show structural details of the arrangement of α-helices, β-sheets and turns of each finger as well as the potential location in them of the Zn^2+^ ion. In addition, the relative distribution of these structures in all Zbed4 and Zbed1 fingers can be observed in the superimposed image of the models (Fig. 10B), which shows that all of the BED zinc-fingers have a very close 3D structure. To determine if Zbed4 is a sequence-specific binding protein, we used CASTing. For these experiments, a pool of random oligonucleotides was incubated with recombinant Zbed4. Following several rounds of selection and amplification of the DNA segments binding to Zbed4, these were cloned and sequenced. The majority of the clones contained poly-G tracts, indicating that these might be Zbed4's binding sequences. To verify that these were the specific nucleotides that interact with Zbed4, we performed electrophoretic mobility shift assays. These assays are usually carried out on native polyacrylamide gels [32], [40], [41] **.** In our experiments, the complexed Zbed4-DNA was stacked at the top of the gel, probably because of its high molecular weight, charge or aggregation state. We were able to overcome this difficulty using 1% agarose gel buffered by 25 mM HEPES, pH 8.2. The high intensity of the retarded DNA band stained by SYBR Gold confirmed a strong DNA-Zbed4 interaction (Fig. 5A). Together, our CASTing and EMSA results demonstrated that Zbed4 has strong affinity for poly-G sequences in double- and single-stranded DNA. Poly-G tracts must contain 5 or more Gs in order for Zbed4 to bind to them.

We also investigated whether Zbed4 interacts with RNA with the use of poly-rC and poly-rG oligonucleotides in the binding reactions. We found that only poly-rG interacted with Zbed4, confirming once more, the high affinity of Zbed4 for poly-G, being it either deoxy- or riboxy-nucleic acid. Since studies in the literature have shown that zinc-finger proteins can bind not only to single-stranded RNA but also to double-stranded RNA [42], we can hypothesize that Zbed4 will interact with double-stranded RNA too, and even assume that it will do the same with DNA-RNA hybrids.

To determine whether Zbed4 also interacts with G-rich elements such as the GC-box, we analyzed its binding to two variants of the GC box consensus sequence, G**GGGCGG**GGC and T**GGGCGG**AAT, both having the same core (bolded). The GC box is one of the most common regulatory DNA elements in the promoter region of eukaryotic genes [43]. Zbed4 only interacted with GGGGCGGGGC, suggesting that the neighboring nucleotides of the core are crucial.

One of the goals of the present study was to search for binding partners of Zbed4 and here we report the identification of SAFB1, ERα and MYH9 as Zbed4-interacting proteins expressed in Y79 retinoblastoma cells.

Zbed4-SAFB1 close association was first identified by immunoprecipitation with Zbed4 antibody from Y79 retinoblastoma cell extracts and mass spectrometry of the separated proteins and was confirmed *in vitro* with a Zbed4 membrane overlay assay and *in vivo* by the presence of both proteins in the Zbed4 and SAFB1 co-immunoprecipitates from the same cell extracts. Moreover, Zbed4 and SAFB1 co-localized to the nuclei of Y79 retinoblastoma cells, providing further evidence of their interaction *in vivo*. SAFB1 is a large protein with multiple functions that include transcriptional regulation, RNA splicing and RNA metabolism [44]. Recent studies suggest its role in chromatin reorganization and demonstrate that SAFB1 is involved in various cellular processes like cell growth, stress response and apoptosis [45] **.** SAFB1 also functions as an ERα co-repressor [44], [45]. Based on our results on Zbed4 and SAFB1 interaction and the fact that co-repressors regulate receptor activity by forming multi-protein complexes, we speculate that both proteins may be components of one of these complexes that could lead to chromatin remodeling, competition with co-activators, and interference with DNA binding**.**

Our results also demonstrate the nuclear and cytoplasmic co-localization of Zbed4 and ERα in Y79 retinoblastoma cells and they confirm the close association of these proteins. Estrogen receptors are regarded to be cytoplasmic receptors in their unliganded state but a fraction of the receptors reside in the nucleus [46] **.** ERα works through different pathways to stimulate transcription of estrogen-responsive genes [47]. It is interesting that Sp1 sites can also mediate estrogen induction via ERα in the context of some promoters, but ERα does not bind to these sites and regulation occurs through protein-protein interactions between the transcription factors and ERα. We hypothesize that Zbed4 may function in a similar way to Sp1 on the promoter of retinal genes, since both transcription factors have zinc-fingers and bind to G-rich sequences. Moreover, Zbed4 may also interact indirectly with ERα, as a co-regulator with SAFB1.

In addition to SAFB1 and ERα, we show here that Zbed4 interacts with another protein, Myosin 9 (MYH9), also known as Myosin IIA. This protein is arranged in bundles of filaments in the cytoplasm of non-muscle cells, where it is thought to be responsible for vesicle formation and movement. MYH9 has been localized to the trans-Golgi network and is probably a key player in protein and vesicle trafficking to and from the Golgi apparatus [48]. We speculate that in Y79 retinoblastoma cells as well as in retina Zbed4 may be transported by the MYH9 trafficking system.

Most importantly, we have identified one of many possible functions of Zbed4: it acts as a transcription factor. Our results show that Zbed4, expressed in stably-transfected HEK293 cells, was able to increase luciferase gene expression driven by different retinal promoters containing poly-G tracts above the expression level observed with non-transfected HEK293 cells, used as control. The vimentin reporter plasmid was the most stimulated by Zbed4, probably because it has two 5-G tracts compared to the other three promoters that only have one poly-G tract. Furthermore, as we showed in our *in vitro* experiments (Fig. 6), the number of Gs in the tract is important for Zbed4 binding. This also may occur *in* *vivo*, and could explain why the rhodopsin promoter, with a single 8-G tract, is activated by Zbed4 more than the blue opsin promoter that has 6 consecutive Gs, which in turn binds Zbed4 better than the green opsin promoter with only 5Gs. As expected, the α-PDE promoter carrying no poly-G tracts was not activated by Zbed4. The small, but statistically significant increase in luciferase gene expression observed for all the other promoters, might be due to the absence in HEK293 cells of additional proteins that are important for the proper arrangement of the transcriptional complex**.** Supporting this hypothesis, we had previously demonstrated that the photoreceptor β-PDE promoter is specifically trans-activated by Sp4, but that this activation increases many-fold when Sp4 interacts with other transcription factors such as Nrl, Crx and TFIIB [49], [50] **.** A similar scenario has been reported by Wu and colleagues [51] who demonstrated that the SmNR1 protein alone is able to activate the transcription of a reporter gene in COS-7 cells, but when another protein already known to interact with it, SmRXR1, is present, SmNR1’s activation increases approximately 2-fold. As for Zbed4 in the current study, if a binding partner were present in the HEK293 cells, the increase in the transcriptional activation of the promoters analyzed would probably be much more substantial. In order to better characterize Zbed4’s action as a transcription factor, future experiments designed to determine whether SAFB1 and ERα (or other nuclear proteins interacting with Zbed4) are present and active in HEK293 cells will be necessary.

In conclusion, we successfully expressed a His-tagged Zbed4 fusion protein in *E. coli*, and developed an optimized purification and refolding procedure for obtaining milligram amounts of homogeneous recombinant, active protein. Initial structural analysis revealed that the primary organization of Zbed4 has an α-helix content that is typical for most zinc-finger proteins [37]. We showed that Zbed4 has DNA- and RNA-binding activity and that it specifically interacts with G-rich sequences. Our experiments also confirmed the nuclear co-localization and possible association of ZBED4 with SAFB1 and ERα and the cytoplasmic interaction of Zbed4 with MYH9 in Y79 retinoblastoma cells. A major finding of this work is the demonstration of the ability of Zbed4 to activate gene transcription. Future studies based on the results presented here will establish the specific role and potential importance of Zbed4 in retinal function.

## Materials and Methods

### Ethics Statement

All experiments involving mice were carried out using protocols approved by the UCLA Animal Research Committee, and in accordance with the ARVO Statement for Use of Animals in Ophthalmic and Vision Research. The cell lines used were obtained from commercial vendors: BL21(DE3)Star cells from Invitrogen (Grand Island, NY); Y79 retinoblastoma and HEK293 cells from ATCC;

### Bacterial Strains, Plasmids and Oligonucleotides

Bacterial strains and eukaryotic cell lines as well as all plasmids used in this study are listed in Table S1. All oligonucleotides were designed using the DNASTAR 5.0 software package and Amplify 1.2, in an effort to minimize non-specific DNA amplification (Table S3).

### Construction of the Zbed4 Plasmid for Expression in E. coli Cells

A 3555 bp fragment containing the coding region of Zbed4 and 8 extra histidines at its 3' end was amplified by RT-PCR from mouse retinal RNA using Pfu-Ultra DNA-polymerase (Agilent Technologies, Santa Clara, CA) and primers ZB4NheI-F and ZB4His8BamHI-R (Table S3). The PCR product was then purified with the GeneElute Gel Extraction kit (Sigma-Aldrich Corporation, St Louis, MO), digested with NheI and BamHI, and cloned into the NheI–BamHI-restricted *E. coli* expression vector pET11a+. This expression plasmid was designated as pZbed4complete and its correct sequence was confirmed on a Biosystems 3730 Capillary DNA Analyzer (UCLA Genotyping and Sequencing Core).

### Zbed4 Expression, Purification and Refolding

*E. coli* BL21(DE3)Star cells were transformed with pZbed4complete and pLysSRARE2, plated on solid LB-buffered agar [1% (w/v) tryptone (Difco, Franklin Lakes, NJ), 0.5% (w/v) yeast extract (Difco), 1% (w/v) NaCl, 50 M Na_2_HPO_4_/NaH_2_PO_4_, 2.0% (w/v) agar (Difco)], pH 7.5, containing 100 µg/ml ampicillin, 30 µg/ml chloramphenicol and incubated at 37°C. An individual bacterial colony was inoculated in 100 ml of medium with 100 µg/ml ampicillin, 30 µg/ml chloramphenicol. This pre-culture was grown overnight at 37°C with continuous shaking at 200 rpm and then used to inoculate 1 L of buffered LB medium (same ingredients as above, without the agar). The culture was grown for about 2.5 h at 37°C to an OD_600_ 0.6, and 0.5 mM IPTG (final concentration) was added to induce the expression of protein. Growth was continued for 6 h with shaking at 200 rpm. Cells were then harvested: about 2.5 g of wet packed cells were obtained per liter of culture. Cells were suspended in 20 ml of 20 mM HEPES buffer, pH 7.5, containing 0.5 M NaCl, 1% Triton X100, 5% glycerol, 1× Complete Protease Inhibitor Cocktail (Roche, Indianapolis, IN) and a mixture of 10 µg DNaseI, 10 µg RNaseA and 50 µg lysozyme, and were disintegrated by passing through a French pressure chamber twice at 82.7 MPa. The suspension was centrifuged at 4°C for 1 h at 150,000 g. The inclusion bodies-containing pellets were resuspended in urea washing buffer (20 mM HEPES, pH 7.5, 0.5 M NaCl, 8 M urea) and centrifuged using the same conditions. Pellets were dissolved in 25 ml of Guanidine HCl buffer 1 (6 M Gu-HCl, 20 mM HEPES, pH 7.5, 0.5 M NaCl, 5 mM imidazole, 4 mM β-mercaptoethanol) by overnight incubation at 4°C while rotating. The mixture was centrifuged for 1 h at 150,000 g at 4°C, and the supernatant was applied to a column with Co^2+^-activated BD TALON metal affinity resin (Clontech, Mountain View, CA) equilibrated in Gu-HCl buffer 1. The column was washed with 20 column bed volumes of Gu-HCl buffer 2 (6 M Gu-HCl, 20 mM HEPES, pH 7.5, 0.5 M NaCl, 20 mM imidazole, 4 mM β-mercaptoethanol). Protein was eluted with Gu-HCl elution buffer (6 M Gu-HCl, 20 mM HEPES, pH7.5, 0.5 M NaCl, 200 mM imidazole, 100 mM EDTA, 4 mM β-mercaptoethanol). Eluted protein was dialyzed against refolding buffer (20 mM HEPES, pH 7.5, 150 mM NaCl, 5 mM β-mercaptoethanol, 5% glycerol, 0.5 mM L-ascorbic acid, 0.5 mM propyl gallate, 10 µM ZnSO_4_) and concentrated using a 15-ml Amicon Ultra concentrator (Millipore, Billerica, MA).

### SDS-PAGE and Western Blots

50 µg protein from each fraction of the purification/refolding steps were electrophoresed on 4–12% Bis-Tris gels (Invitrogen) and the separated proteins were electro-transferred to PVDF membranes, which were subsequently blocked with 3% (w/v) casein. Blots were incubated with rabbit Penta-His antibodies conjugated with horseradish peroxidase (1∶1000 dilution, Qiagen, Valencia, CA). Western blots were visualized with the ECL Substrate of the Fast Western blot kit (Thermo Scientific Pierce, Rockford, IL).

### Circular Dichroism (CD) Spectroscopy of Purified Zbed4

Before CD spectra analyses, Zn^2+^-bound Zbed4 was dialyzed against 20 mM HEPES, pH 7.5, 150 mM NaCl, and 5% glycerol. Far-UV CD measurements were performed at 22°C with a JASCO J-810 spectropolarimeter (JASCO, Easton, MD) and spectra were acquired from 16 scans between 260 to 195 nm in 0.2 mm path length cells. A 0.4 mg/ml Zbed4 protein sample was used. CD spectra were corrected for buffer contributions. Ellipticity is reported as mean molar residue ellipticity (MRE) in degree.cm^2^.dmol^−1^. Protein concentration was determined using the MicroBCA protein assay kit (Thermo Scientific).

### Determination of Zbed4 Binding Sites with CASTing Experiments

Double-stranded degenerate fragments were amplified by PCR from oligonucleotide CASTrandom (Table S3). The PCR reaction mixture (50 µl) contained 1× ThermoPol buffer (New England Biolabs, Ipswich, MA), 1 pmol of each primer (CAST-F, CAST-R, Table S3), each nucleotide at a final concentration of 0.25 mM, 10% (v/v) dimethyl sulfoxide, 1 U Taq-polymerase (New England Biolabs) and 1 pmol CASTrandom. Binding reactions were performed by adding Zbed4 (20 µg) in refolding buffer to the double stranded degenerate oligonucleotide (1.8 ng) and incubating at room temperature for 30 min. Then, Zbed4 antibody (20 µg) and Protein A Sepharose beads (20 µl) were added and incubation continued at room temperature for an additional 1 h. The DNA-protein complexes formed were precipitated by centrifugation at 1000 g, and the pellets were washed 6 times with refolding buffer. Bound DNA was extracted with phenol/chloroform and amplified by 35 cycles of 1 min at 94°C, 30 sec at 55°C, and 1 min at 72°C in the ThermoPol PCR system (New England Biolabs). The series of steps of incubation, immunoprecipitation, and re-amplification, considered as one cycle of CASTing, were repeated three times. The PCR product was cloned into the pCR2.1-TOPO plasmid. Nucleotide sequences of 40 independent clones were determined on the Biosystems 3730 Capillary DNA Analyzer (UCLA Genotyping and Sequencing Core).

### Electrophoretic Mobility Shift Assay (EMSA)

Protein–DNA binding reactions contained Zbed4 (20 µg) and 0.5 nM oligonucleotide in refolding buffer. Reaction mixtures were incubated at room temperature for 45 min and then were loaded on 1% agarose gels pre-electrophoresed for 1 h. Gels were run at room temperature in 25 mM HEPES buffer, pH 8.2, at 20 mA, and were stained with the SYBR Gold nucleic acid stain solution (Invitrogen) for 10 min in the dark. The fluorescence of the gel bands was visualized under UV light. The agarose gels were subsequently stained with Coomassie R-250 for detection of Zbed4.

### Cell Culture

Y79 retinoblastoma cells were cultured using RPMI medium (Sigma) supplemented with 15% fetal bovine serum, and human embryonic kidney (HEK293) cells were cultured in DMEM medium supplemented with 13% FBS; both cell lines grew at 37°C in a humidified incubator with 95% air and 5% CO2.

HEK293 cells were used for the generation of a stable transfected cell line expressing Zbed4 [14]. Briefly, gel-purified Zbed4 cDNA amplified by RT-PCR from mouse mRNA was subcloned into the eukaryotic expression vector pcDNA4/HisMax (Invitrogen) containing the Zeocin resistance gene. Transfection was performed with Superfect Transfection Reagent (Qiagen) and Zeocin resistant transformants were selected 4 weeks after transfection.

### Zbed4 Immunostaining in Y79 Retinoblastoma and Stably Transfected HEK293 Cells

Cultured cells (6×10^5^) were seeded on poly-D-lysine and fibronectin-coated cover slips and allowed to adhere at least for 24 h at 37°C before immunostaining. Cells were then fixed with ice-cold methanol at −20°C and washed with 1×PBS. After blocking with 2% BSA, 0.1% Triton X-100 in 1×PBS for 40 min, rabbit polyclonal Zbed4 antibody ([16], 1∶300 dilution) was applied to the slides and the cells were incubated for 2 h at room temperature, followed by three washes in 1×PBS, 0.1% Triton X-100 and incubation with secondary anti-rabbit goat antibodies labeled with Alexa-488 (Invitrogen, 1∶300 dilution) for 1 h at room temperature. After subsequent washes in 1×PBS, 0.1% Triton X-100, nuclei were stained with DAPI for 5 min at room temperature. Slides were mounted and viewed on an Olympus Fluoview FV 1000 Laser scanning microscope (Olympus America, Center Valley, PA).

### Co-localization of Zbed4 with SAFB1, ERα or MYH9

Similar immunocytochemistry experiments were performed using Y79 retinoblastoma cells to detect co-localization of Zbed4 with SAFB1, ERα, or MYH9 using rabbit ZBED4 (1∶300), goat SAFB1 [(Bethyl Laboratories, Montgomery, TX), 1∶200], mouse ERα [(Abcam, Cambridge, MA), 1∶100] or rabbit MYH9 (Abcam, 1∶400) primary antibodies along with goat anti-rabbit Alexa-488 for Zbed4 (1∶1000 dilution) and donkey anti-goat Alexa-568 (1∶1000 dilution), goat anti-mouse Alexa-568 (1∶1000) or goat anti-rabbit secondary antibodies (Invitrogen) for SAFB1, ERα or MYH9, respectively.

### Immunoprecipitation and Mass Spectrometry

Y79 cells were lysed using RIPA lysis buffer (150 mM NaCl, 1% IGEPAL CA-630, 0.5% sodium deoxycholate, 0.1% SDS, 50 mM Tris, pH 8.0, and 1 mM PMSF) and their proteins were immunoprecipitated with the Zbed4 antibody following the Immobilized Protein G kit protocol (Pierce). As a control, the same reaction was carried out using pre-immune serum. The precipitated proteins were separated on SDS-PAGE and stained with SYPRO Ruby. Protein bands were excised and destained for *in gel* trypsin digestion. The digested bands were subjected to nLC/MS/MS mass spectrometry (MDS Sciex QSTAR XL Quad - Time-of-Flight Mass Spectrometer, Applied Biosystems, Carlsbad, CA) at the UCLA’s Pasarow mass spectrometry laboratory and data were analyzed with the Sequest database search algorithm implemented in the proteome Discoverer software (Thermo Fisher, Waltham, MA).

### Gel Overlay Assay

Nuclear extracts from Y79 retinoblastoma cells were subjected to SDS-PAGE and the separated proteins were transferred to PVDF membranes. After blocking with Odyssey blocking buffer (LI-COR, Lincoln, NE) for 1 h at room temperature, the blots were incubated for 2 h at 5°C in the dark with purified Zbed4 protein (5–10 µg/ml) in 10 mM Tris-HCl (pH 8), 150 mM NaCl, and 0.05% Tween-20. The membranes were washed with 1× PBS and 0.1% Tween-20 and incubated separately with rabbit anti-Zbed4 (1∶1000), goat anti-SAFB1 (1∶500), and rabbit anti-ERα (1∶100) antibodies for 1 h, washed with 1× PBS and 0.1% Tween-20, and then incubated with IRDye 800 CW goat anti-rabbit (1∶15,000) and IRDye 700 DX donkey anti-goat (1∶15,000) secondary antibodies (LI-COR) for 1 h. Membranes were then washed and scanned on the LI-COR Odyssey Infrared imager.

### Co-immunoprecipitation of Zbed4 with SAFB1 or MYH9

Proteins of cultured Y79 retinoblastoma cells immunoprecipitated as described above using rabbit Zbed4, goat SAFB1 or rabbit MYH9 antibodies were separated on triplicate lanes of 4%–20% Pierce Precise Protein gels (Thermo-Scientific) by electrophoresis, using Tris-Glycine-SDS buffer at 70 volts. Proteins were then transferred to PVDF membranes, which were blocked with 3% BSA in Tris Base Tween (TBST) buffer, pH 7.5, for 1 h. Proteins precipitated with the Zbed4 antibody were probed on the Western with SAFB1 or MYH9 primary antibodies and those precipitated with the SAFB1 or MYH9 antibodies were probed with the Zbed4 primary antibody. The remaining membrane with proteins of each immunoprecipitation was incubated with the same antibody used for bringing down the proteins. Then, anti-rabbit alkaline phosphatase antibody was added to each of the samples followed by application of DuoLux Chemiluminescent/Fluorescent Substrate for alkaline phosphatase (Vector Laboratories, Burlingame, CA). After washing with buffer, the membranes were exposed to film (GE Healthcare, Piscataway, NJ).

### Transient Transfections and Reporter Assays

Reporter plasmids containing 5'-flanking regions of the genes encoding mouse vimentin and human blue and green opsins were cloned into the pGL2 Basic vector (Promega, Madison, WI) that contains the luciferase gene. These plasmids were called pVIM/*luc*, pBLUE/*luc* and pGREEN/*luc*, respectively (Table S1).

For transient transfection assays, 3×10^6^ stably-transfected HEK293 cells expressing Zbed4 and HEK293 non-transfected cells (used as control) were plated on 60 mm culture plates in Dulbecco’s modified Eagle’s medium/Ham’s F-12 (Invitrogen) supplemented with 10% fetal bovine serum 24 h prior transfection. Calcium phosphate-mediated transient transfections of pVim/*luc*, pRho/*luc*, pBlue/*luc*, pGreen/*luc*, pα-PDE/*luc* and pGL2 Basic vector as well as luciferase and β-galactosidase assays were performed as described previously [52], except for the addition of a glycerol shock that improved the transfection efficiency of HEK293 cells. The glycerol shock was performed after 5 h of exposure of the cells to the calcium phosphate-DNA co-precipitate at 37°C under 5% CO_2_. For this, the cells were washed with 1× PBS and overlayed with 15% glycerol in 1× PBS for 45 sec at room temperature, washed with 1× PBS and kept in DMEM with 10% fetal bovine serum for 48 h before luciferase and β-galactosidase assays. Triplicate plates were used for transfection of each reporter construct. The Promega Luciferase Assay System (Promega) was used for measuring luciferase activity following the manufacturer’s instructions. Luciferase activity produced by each reporter plasmid was normalized for each transfection system, using the β-galactosidase assay. Relative luciferase activity was calculated as luciferase light units, divided by β-galactosidase activity x 1000.

## Supporting Information

Table S1 Bacterial strain, eukaryotic cell lines and plasmids used in these studies.
(DOCX)

Table S2 Secondary structure content for Zbed4 at pH 7.3 determined from its CD spectrum.
(DOCX)

Table S3 List of primers used throughout these studies.
(DOCX)
