# Supplementary material for: Sequence-Specific Binding of Recombinant Zbed4 to DNA: Insights into Zbed4 Participation in Gene Transcription and Its Association with Other Proteins
Source: PLoS One. 2012 May 31;7(5):e35317. doi: 10.1371/journal.pone.0035317 (PMC3365051; doi:10.1371/journal.pone.0035317)
Supplement: Table S1 — Bacterial strain, eukaryotic cell lines and plasmids used in these studies. (DOCX) [file pone.0035317.s001.docx]

**Table S1.**

|  | Description | Source |
| --- | --- | --- |
| ***Bacterial strains***  DH5α  BL21(DE3)  BL21(DE3)Star  B834(DE3)  C41(DE3)  C43(DE3)  ***Eukaryotic Cell Lines***  HEK293  Zbed4-stably-transfected HEK293  Y79 retinoblastoma cells  ***Plasmids***  pLysSRARE2  pET11a+  pCR®2.1-TOPO®  pZbed4complete  pcDNA4/HisMAX  pGL2basic  pSV-β-Galactosidase  pZbed4eu  pVIM/*luc*  pBLUE/*luc*  pGREEN/*luc*  pRHO/*luc*  pα-PDE/*luc* | F^-^ endA1 glnV44 thi-1 recA1 relA1 gyrA96 deoR nupG Φ80d*lacZ*ΔM15 Δ(*lacZYA-argF*)U169, hsdR17(r_K_^-^ m_K_^+^), λ–  F– ompT hsdSB(rB–, mB–) gal dcm (DE3)  F– ompT hsdSB(rB–, mB–) gal dcm rne131 (DE3)  F– *ompT hsdS*B(rB–, mB –) *gal dcm met* (DE3)  BL21(DE3) with uncharacterized mutation that occurred by a spontaneous process  Double mutant strain was selected from C41(DE3) cells  Human Embryonic Kidney cells  Human Embryonic Kidney cells stably transfected by pZBED4eu  Human Y79 retinoblastoma cells  Plasmid containing tRNA genes for rare codons (AUA, AGG, AGA, CUA, CCC, CGG, GGA) in *E. coli* and the gene encoding T7 lysozyme  E. coli expression plasmid under control of T7 regulatory elements  Cloning plasmid  Plasmid for Zbed4 expression under control of T7 regulatory elements  Eukaryotic expression plasmid under control of the CMV promoter  Reporter plasmid for cloning the promoter region  Control vector for monitoring transfection efficiencies  Plasmid for stable expression of ZBED4 in eukaryotic cells  Vimentin/luciferase reporter plasmid (5’-flanking region, -1190 +1)  Blue opsin/luciferase reporter plasmid (5’-flanking region, -1190 +1)  Green opsin/luciferase reporter plasmid (5’-flanking region, -276 +79)  Rhodopsin/luciferase reporter plasmid (5’-flanking region, -130 to +70)  PDE6A/luciferase reporter plasmid (5’-flanking region, -132 to +139) | Invitrogen  Invitrogen  Invitrogen  Novagen  Avidis SA  Avidis SA  ATCC  From our lab [11]  ATCC  Novagen  Novagen  Invitrogen  This study  Invitrogen  Promega  Promega  From our lab [11] This study  This study  This study  [8]  This study |
