# Supplementary material for: Sequence-Specific Binding of Recombinant Zbed4 to DNA: Insights into Zbed4 Participation in Gene Transcription and Its Association with Other Proteins
Source: PLoS One. 2012 May 31;7(5):e35317. doi: 10.1371/journal.pone.0035317 (PMC3365051; doi:10.1371/journal.pone.0035317)
Supplement: Table S2 — Secondary structure content for Zbed4 at pH 7.3 determined from its CD spectrum. (DOCX) [file pone.0035317.s002.docx]

## Table S2.

| Protein | Program | α- helix (%)^a^ | | |  | β- strand (%)^b^ | | |  | turn  (%) | unrd^c^  (%) | nrmsd | |
| --- | --- | --- | --- | --- | --- | --- | --- | --- | --- | --- | --- | --- | --- |
|  |  | H(r) | H(d) | ∑H |  | S(r) | S(d) | ∑S |  |  |  |  |  |
| Zbed4 | CONTINLL | 17.0 | 14.2 | 31.2 |  | 9.7 | 7.9 | 17.6 |  | 21.9 | 29.2 | 0.018 | |
|  | SELCON3 | 18.4 | 14.0 | 32.4 |  | 10.0 | 7.7 | 17.7 |  | 20.0 | 29.9 | 0.349 | |
|  | CDSSTR | 20.0 | 14.9 | 34.9 |  | 10.3 | 7.4 | 17.7 |  | 18.2 | 29.7 | 0.080 | |
| ^a^ H(r) and H(d): regular and distorted α-helix, respectively. ∑H= H(r) + H(d)  ^b^ S(r) and S(d): regular and distorted β-strand, respectively. ∑S= S(r) + S(d).  ^c^ unrd: unordered fraction which contains residues that are not assigned to any defined structural class.  Nrmsd: normalized root-mean-square deviation. | | | | | | | | | | | | |  |
