# Supplementary material for: Sequence-Specific Binding of Recombinant Zbed4 to DNA: Insights into Zbed4 Participation in Gene Transcription and Its Association with Other Proteins
Source: PLoS One. 2012 May 31;7(5):e35317. doi: 10.1371/journal.pone.0035317 (PMC3365051; doi:10.1371/journal.pone.0035317)
Supplement: Table S3 — List of primers used throughout these studies. (DOCX) [file pone.0035317.s003.docx]

## Table S3.

| Primer sequence (5’-3’) | Name | Primer sequence (5’-3’) | Name |
| --- | --- | --- | --- |
| Cloning | | | |
| 1. TTTTTGCTAGCATGGAGGATAAGCA- AGAAACTTGTCCCAAG  2. TTTTTGGATCCTTAATGGTGATGGTG- ATGGTGATGGTGATACTGAAAGCATA -TTAAAGGAAGATTC  3. TTTTTGGTACCCCTTTCTTCCGAACT- CATTCACCG  4. TTTTTGCTAGCGACTGGACAACGCC- AAGGGCGCTG | ZB4NheI-F  ZB4BamHI-R  VIM-F  VIM-R | 5. TTTTTGGTACCATCTATATATATAGTCTCGC- TCTGTC  6. TTTTTGCTAGCCACCCCCCTCTGAGTCCTC- TTATAG  7. TTTTTGGTACCAGACAGAGTCTTGGTCTG-TTGC  8. TTTTTGCTAGCTATGGAAAGCCCTGTCCC- CGAC | BLUE-F  BLUE-R  GREEN-F  GREEN-R |
| CASTing | | | |
| 9. CAGGTCAGTTCAGCGGATCCTGTCG- NNNNNNNNNNNNNNNNNNNNNNNN- NNAGGCGAATTCAGTGCAACTGCAGC | CASTrandom | 10. GGTCAGTTCAGCGGATCCTGTC  11. GCTGCAGTTGCACTGAATTCGC | CAST-F  CAST-R |
| EMSA | | | |
| 12. AAAAAAAAAAAAAAAAAAAA  13. CCCCCCCCCCCCCCCCCCCC  14. GGGGGGGGGGGGGGGGGGGG  15. TTTTTTTTTTTTTTTTTTT  16. YYYYYYYYYYYYYYYYYYYY  17. WWWWWWWWWWWWWWWWWWWW  18. MMMMMMMMMMMMMMMMMMMM  19. RRRRRRRRRRRRRRRRRRRR  20. SSSSSSSSSSSSSSSSSSSS  21. KKKKKKKKKKKKKKKKKKKK  22. NNNNNNNNNNNNNNNNNNNN  23. AAAAAGGGGCGGGGCAAAAA  24. AAAAATGGGCGGAATAAAAA  25. AAAAAAAAGGGAAAAAAAAA  26. AAAAAAAAGGGGAAAAAAAA  27. AAAAAAAGGGGGAAAAAAAA | A20  C20  G20  T20  Y20  W20  M20  R20  S20  K20  N20  GC-box1  GC-box2  G3  G4  G5 | 28. AAAAAAGGGGGGAAAAAAAA  29. AAAAAAGGGGGGGAAAAAAA  30. AAAAAAGGGGGGGGAAAAAA  31. AAAAAAGGGGGGGGGAAAAA  32. AAAAAGGGGGGGGGGAAAAA  33. AAAAAGGGGGGGGGGGAAAA  34. AAAAGGGGGGGGGGGGAAAA  35.AAAGGGGGGGGGGGGGAAAA  36. AAAGGGGGGGGGGGGGGAAA  37. AAGGGGGGGGGGGGGGGAAA  38. AAGGGGGGGGGGGGGGGGAA  39. AAGGGGGGGGGGGGGGGGGA  40. AGGGGGGGGGGGGGGGGGGA  41. GGGGGGGGGGGGGGGGGGGA  42. UUUUUrGrGrGrGrGrGrGrGrGrGUUUUU  43. UUUUUrCrCrCrCrCrCrCrCrCrCUUUUU | G6  G7  G8  G9  G10  G11  G12  G13  G14  G15  G16  G17  G18  G19  rG10  rC10 |
| According to the IUPAC nucleotide ambiguity codes, A=adenine, C=cytosine, G=guanine, T=thymine, U=uracil, K=G or T, M=A or C, N=A or C or G or T, R=A or G, S=C or G, W=A or T, Y= C or T. | | | |

## 
